# Optical Patterning of Two-Dimensional Materials

**DOI:** 10.34133/2020/6581250

**Published:** 2020-01-27

**Authors:** Pavana Siddhartha Kollipara, Jingang Li, Yuebing Zheng

**Affiliations:** ^1^Walker Department of Mechanical Engineering, The University of Texas at Austin, Austin, TX 78712, USA; ^2^Materials Science & Engineering Program and Texas Materials Institute, The University of Texas at Austin, Austin, TX 78712, USA

## Abstract

Recent advances in the field of two-dimensional (2D) materials have led to new electronic and photonic devices enabled by their unique properties at atomic thickness. Structuring 2D materials into desired patterns on substrates is often an essential and foremost step for the optimum performance of the functional devices. In this regard, optical patterning of 2D materials has received enormous interest due to its advantages of high-throughput, site-specific, and on-demand fabrication. Recent years have witnessed scientific reports of a variety of optical techniques applicable to patterning 2D materials. In this minireview, we present the state-of-the-art optical patterning of 2D materials, including laser thinning, doping, phase transition, oxidation, and ablation. Several applications based on optically patterned 2D materials will be discussed as well. With further developments, optical patterning is expected to hold the key in pushing the frontiers of manufacturing and applications of 2D materials.

## 1. Introduction

Nanomaterials, with at least one geometric dimension at the nanometer scale, have immense applications in miniaturization of existing technology while rendering the intense exploration of fundamental phenomena at nanoscale. 2D materials are a subset of the nanomaterials where geometric length of only one dimension reaches the nanometer regime [1]. Since its inception in 2004 [2], graphene has become one of the most interesting 2D materials that has been extensively studied for its excellent physical properties such as visual transparency [3], high mechanical strength [4], and extraordinary electrical and thermal conductivities [5, 6]. Graphene has reigned the 2D material community for more than a decade, but has been gaining an ever-increasing competition from other 2D materials and their van der Waals heterostructures such as hexagonal boron nitride (hBN), phosphorene, and transition metal dichalcogenides (TMDCs) [7]. These emerging 2D materials fundamentally differ from graphene in their physical and chemical compositions, which increases the functional diversity of 2D materials and devices. Researchers have been consistently exploiting a variety 2D materials to enhance the performance of energy storage [8], transistors [9–12], Hall effect sensors [13], flexible electronics [14, 15], optical modulators [16], and thermal management [17, 18].

For application-oriented utilization of 2D materials, one often starts with their fabrication or patterning, which might involve control of either thickness, carrier density, or phase of 2D materials to precisely tune the optical, electrical, and thermal characteristics of 2D materials. The patterned 2D materials have led to applications in electrochemistry [19], biosensing [20], solar cells [21], infrared detection [22], and optical spectroscopy [23]. A variety of methods were employed to achieve patterning of 2D materials. The conventional electron beam lithography (EBL) has been widely exploited to pattern 2D materials with nanometer scale features [24, 25]. However, challenges such as high cost of instruments, high vacuum, complex operation, and low throughput significantly limit the use of EBL. Apart from these challenges, EBL introduces inadvertent impurity doping along with dramatic changes to structural integrity [26, 27]. Other methods such as thermal annealing [28] and plasma treatment [29] were demonstrated to achieve precise control over layer-by-layer thinning of 2D materials. However, thermal annealing is temperature-dependent and takes several hours to alter the thickness for tuning the electrical properties [30]. Execution of plasma etching has been difficult due to the challenge in identification of crystallographic orientation of patterned materials [31, 32]. Therefore, better universal techniques to achieve lateral and out-of-plane patterning of 2D materials with improved throughput are highly desired for their diverse applications.

Laser-based fabrication methods provide a promising route to high-throughput, versatile, and *in situ* patterning of 2D materials through precise digital control of laser beams. The localized nature of high-intensity laser beams offers unique opportunities to develop or pattern 2D materials based on regionally induced physical or chemical modifications. For many micro/nanodevice applications of 2D materials based on their patterned features at micro/nanoscale, the timescales of optical patterning range from a few seconds to minutes. Moreover, laser intensity can be tuned with ease to peel a desired number of atomic layers from the 2D materials [33] and to control the targeted formation of any desired defects in the 2D materials [34]. Recent years have witnessed rapid progress in optical patterning of 2D materials due to these clearly distinct advantages. Herein, we review the state-of-the-art optical patterning techniques of 2D materials and categorize them based on different patterning mechanisms: (i) site-specific control over the thickness of 2D materials through laser thinning, (ii) laser-induced doping and phase transition of 2D materials, (iii) oxidation of 2D materials that are selectively excited upon laser irradiation, and (iv) patterning through laser ablation of 2D materials. Both high-power femtosecond laser ablation and low-power continuous wave (CW) laser ablation using plasmon-enhanced optothermal effects will be discussed. Next, we present various device applications that exploit the optical patterning of 2D materials. We conclude this review by outlining some of these challenges and their possible solutions and future opportunities in the field of optical patterning of 2D materials.

## 2. Laser Thinning of 2D Materials

The physical properties of 2D materials are closely dependent on their thickness. For example, it has been reported that 2D materials exhibit thickness-dependent photoluminescence (PL) where PL intensity increases with decreasing thickness [35]. Upon reaching monolayer thickness, TMDCs transition from indirect-bandgap semiconductors into direct-bandgap semiconductors [36]. Mechanical strain in graphene can be increased by 25% from that in graphite, and the strain can be utilized to enhance the electrical mobilities in the 2D material [37]. Therefore, methodologies that can precisely control layer thickness of 2D materials are desired. In this section, we discuss some of promising techniques for laser thinning of 2D materials based on different mechanisms.

### 2.1. Laser Thinning through Oxidation

Initial attempts to control the thickness of graphene using high-power lasers are based on oxidative burning and subsequent reduction of graphene oxide films [38]. The irradiated graphene oxide (GO) gained a temperature increase greater than 500°C, which locally oxidized carbon to form CO and CO_2_. The patterned GO layer was then reduced to graphene via thermal annealing or hydrazine. However, this mechanism usually takes over six hours and must be carried out in a humid atmosphere for water to intercalate between graphene layers. This thinning method could only reach down to three-layer graphene due to the high in-plane thermal conductivity of graphene that reduced the temperature in the perpendicular direction below 500°C.

**Figure 1:**
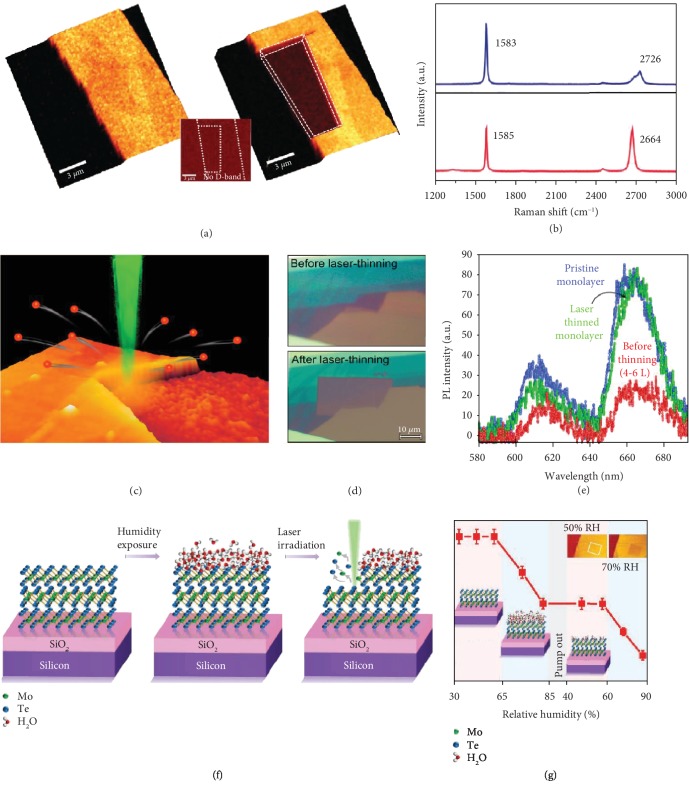
Laser thinning of 2D materials. (a) Raman mapping of G-band before (left) and after (right) laser thinning. The low intensity in the right panel within dashed lines indicates low layer thickness. (b) Raman spectra of graphite before (top) and after (right) laser thinning. Reproduced from Ref. [39] with permission from the American Chemical Society, copyright 2011. (c) Schematic of laser thinning of MoS_2_ via sublimation of top layers. (d) Optical images of MoS_2_ flake before (top) and after (bottom) laser thinning. (e) PL spectra of pristine monolayer MoS_2_, laser-thinned monolayer MoS_2_, and MoS_2_ with 4-6 layers. Reproduced from Ref. [45] with permission from the American Chemical Society, copyright 2012. (f) Mechanistic illustration of humidity-controlled photochemical thinning of MoTe_2_. (g) Flake thickness upon laser scanning as a function of relative humidity. Reproduced from Ref. [48] with permission from Wiley, copyright 2018.

Han et al. proposed that laser thinning of graphite to graphene could be better achieved by similar laser irradiation through substrate engineering [39]. In one of their demonstrations, graphite flakes were introduced on a Si/SiO_2_ substrate that served as a heat sink for the bottom layer of the graphite adjacent to the substrate, thereby reducing the maximum temperature at the bottom layer while the top layers could reach ~1450°C to enable oxidative etching.  Figure 1(a) shows the confocal Raman G-band mapping of a graphite flake before and after laser thinning. The optically etched sample (right panel) has a relatively uniform contrast, indicating that it has uniform thickness. The difference in the layer thickness is quantified by Raman shift of the G′-band (Figure 1(b)), where the peak at 2664 cm^−1^ corresponds to monolayer graphene [40]. The capability of attaining large and uniform monolayer graphene can boost its application in displays because graphene features better transparency than indium tin oxide as the conventional transparent and conductive material [41].

Despite the prominence of monolayer graphene, multilayered graphene exhibits advantages in certain applications. For instance, hydrogenation of multilayered graphene is more feasible than that of monolayer graphene, which is helpful in tuning physical and chemical properties of graphene for sensing devices [42]. Consequently, to precisely control the graphene layers, femtosecond laser pulses with low intensity have been utilized to peel off graphene, layer-by-layer, through repetitive scanning while optically measuring the sample thickness *in situ* through four-wave mixing signals. Although throughput is compromised due to low-power multiple scanning, precise quantification of number of graphene layers has been achieved through *in situ* fast imaging [43].

### 2.2. Laser Thinning through Sublimation

In contrast to graphene whose sublimation temperature is more than 2000°C [44], several 2D materials have a relatively low sublimation temperature that is comparable to their oxidation temperature. For such 2D materials, laser thinning through sublimation is proposed. Castellanos-Gomez et al. have fabricated monolayer MoS_2_ with patterned feature sizes of 200 nm via laser thinning [45]. The laser thinning occurs because of the laser absorption-induced lattice sublimation of top layer of MoS_2_ due to its low sublimation temperature of ~450°C [46] as schematically presented in Figure 1(c). The substrate acts as a heat sink to protect the bottom layer, which however, would also be removed upon increased laser power. The optical contrast of the MoS_2_ flake before and after laser scanning indicates the thinning of the flake (Figure 1(d)). An increase in PL intensity of laser-thinned monolayer MoS_2_ from that of 4-6 multilayered MoS_2_ is observed in Figure 1(e). The PL of the thinned MoS_2_ is comparable to that of pristine monolayer MoS_2_. This PL increase is due to conversion of bulk indirect-bandgap semiconducting MoS_2_ to monolayer direct-bandgap semiconducting MoS_2_ [47]. The contrast in the optical images along with Raman shift and PL can be realized as a feedback mechanism to engineer the thickness of the MoS_2_ flake by tuning the laser power, enabling on-demand fabrication of single-layer/multilayer MoS_2_ with any desired patterns for device applications.

### 2.3. Laser Thinning through Wet Exfoliation

With a direct-bandgap energy of 1.13 eV [9] similar to that of silicon, which extends the spectral response to the near-infrared (NIR) region, MoTe_2_ is favorable for devices such as tunnel field-effect transistors (FETs) and NIR detectors. Recently, Nagareddy et al. have proposed a humidity-controlled laser thinning technique where layer-by-layer laser thinning of MoTe_2_ was achieved for the sample that was maintained at a relative humidity of more than 65-70% [48]. Laser powers much lower than those used for laser thinning through oxidation or sublimation were used because the low bond energies of MoTe_2_ result in an uncontrolled bond-breaking at relatively higher laser powers. As relative humidity increases, the water molecules adsorb on all available sites offered by the top layer of MoTe_2_ (Figure 1(f)). When irradiated with a focused laser beam, the sample undergoes nonlinear optical absorption that results in the generation of excited free electrons, which generate hydroxyl groups from the adsorbed water. The hydroxyl groups attack the holes within MoTe_2_ layers, which breaks Mo-Te bonds. This bond-breaking is initiated at the inherent and stable Te vacancies within the layer and spreads along lateral direction, thereby peeling off the top layer. Since only the top layer is accessible by the hydroxyl groups, laser thinning occurs layer-by-layer with mandatory water adsorption for each laser scan.  Figure 1(g) shows the MoTe_2_ flake thickness upon laser scanning as a function of relative humidity of the ambient atmosphere. Laser scanning at a low power enables continuous thinning of the 2D materials provided the relative humidity is maintained at higher than 65%. Once the humidity is decreased to 40%, no thinning is observed due to low supply of hydroxyl groups that can attack Te vacancies. This thinning mechanism removes the top layer of MoTe_2_ flake without any structural damages caused by optical heating.

## 3. Laser-Induced Doping and Phase Transition of 2D Materials

2D materials offer immense potential in optoelectronics due to active control on charge carrier dynamics [49] and tunable crystal phases. Adjustable free-carrier type, density and mobility in 2D material are important for applications such as transistors [50] and photodetectors [51]. On-demand doping or phase transition of 2D materials at targeted sites has proved effective in enabling desired optical and electronic responses for these applications. In particular, high-intensity laser beams offer several advantages in inducing the site-specific doping or phase transition of 2D materials.

### 3.1. Laser-Induced Doping

**Figure 2:**
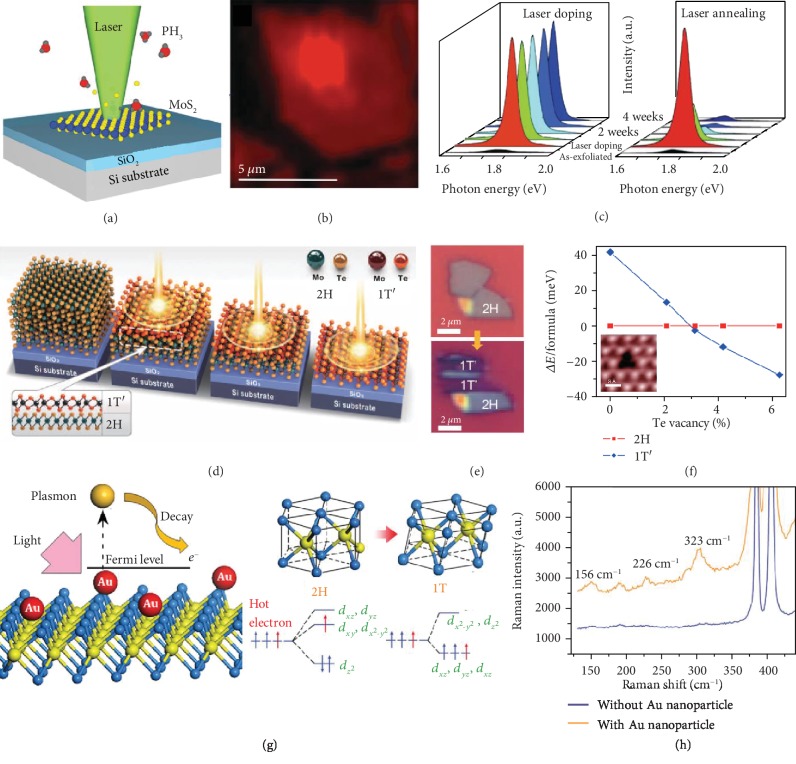
Laser-induced doping and phase transition of 2D materials. (a) Schematic of laser-induced doping of phosphorous into MoS_2_ with phosphine gas. (b) PL mapping of MoS_2_ shows enhanced PL intensity in the phosphorous-doped region. (c) Time-resolved PL spectra show that the sample with laser-induced doping (left) exhibits the higher stability than the sample with laser annealing (right). Reproduced from Ref. [52] with permission from Wiley, copyright 2015. (d) Schematic of laser-induced phase transition of MoTe_2_ from 2H phase to 1T′ phase. (e) Optical images of 2H phase and 1T′ phase of MoTe_2_ before and after laser scanning. (f) Relative binding energy per unit formula for 2H and 1T′ phases as a function of Te vacancy concentration. Inset shows a STEM image of Te vacancy after laser scanning. Scale bar: 3 Å. Reproduced from Ref. [56] with permission from the American Association for the Advancement of Science, copyright 2015. (g) Schematic and mechanism of hot electron-mediated 2H to 1T phase transition in MoS_2_. (h) Raman spectra of MoS_2_ with and without Au nanoparticles showing signature peaks of 1T phase in the presence of Au nanoparticles under laser illumination. Reproduced from Ref. [58] with permission from Wiley, copyright 2014.

Controlled doping of 2D materials is vital for their applications in integrated electronics, photonics, and optoelectronics. To overcome random dopant fluctuations and ensure reproducible device functions, Kim et al. have recently achieved laser-induced doping of phosphorous in monolayer MoS_2_ as illustrated in Figure 2(a) [52]. A 532 nm laser is used to create sulfur vacancies in MoS_2_ flakes through oxidation of sulfur while disassociating phosphine gas into phosphorous ions, which are diffused into MoS_2_ at sulfur vacancies. The PL mapping of the laser-irradiated MoS_2_ flake indicates transition from dark red to bright red due to phosphorous doping (Figure 2(b)). When the laser irradiates the MoS_2_, the induced sulfur vacancies make it *n*-type and increase negative trions over excitons. When MoS_2_ is doped with phosphorous, the trions decrease and excitons dominate because of increased number of holes. As a result, a blue-shift is observed in the PL spectra with the increased intensity in the laser-doped region [53]. To further test the stability of the dopants in MoS_2_, PL spectra of the laser-doped sample were collected each week for a month as shown in Figure 2(c). The intensity of the PL peak did not reduce while the PL spectra from the laser-annealed sample (i.e., laser irradiation in the absence of dopants) exhibited drastically reduced peak intensity within two weeks, indicating that laser-doping is a stable process. The stable PL spectra indicate the applicability of laser beams in site-specific doping and functional tuning of 2D materials for device applications.

### 3.2. Laser-Induced Phase Transition

One of the most prominent challenges in 2D electronics is the reduction of contact resistance between metallic electrodes and semiconducting 2D materials. Several ideas were proposed to reduce the contact resistance, including insertion of conductive graphene layers between the electrodes and the semiconducting 2D materials [54] and chemical doping of 2D materials. Phase transition of 2D materials, which can exhibit shuffling behavior between metallic and semiconducting phases [55], has been another viable approach in tuning the bandgap of the 2D materials and the contact resistance,

Cho et al. identified that transistors made of MoTe_2_ and metallic electrodes suffered from large interfacial resistance and thereby reduced mobility of electrons. To overcome this challenge, they employed a method to transform MoTe_2_ from the 2H to 1T′ phase using a focused laser beam in order to create a heterophase homojunction structure with improved charge carrier mobility and low resistance [56]. The 1T′ phase occurs in octahedral configuration of Mo and Te atoms and has a bandgap of ~60 meV [57], indicating that it is metallic in nature. The 2H phase is semiconducting because of relatively high bandgap (~1.2 eV). Figure 2(d) shows the schematic of the laser-induced phase transition on a 2H MoTe_2_ crystal. As laser irradiation continues, the crystal is thinned due to the 2H-1T′ phase transition that initiates at the top layer. The two distinct phases of MoTe_2_ could be observed from optical contrast in Figure 2(e) and were subsequently substantiated from Raman measurements. The authors claimed that the high-power laser beams increased the temperature of MoTe_2_ to ~400°C and caused the sublimation of Te atoms, thereby creating Te vacancies. The calculated binding energy of 2H and 1T′ phases as a function of Te vacancy concentration proves that Te vacancies greater than 3% would drive effective phase transition of MoTe_2_ to obtain the more stable 1T′ phase (Figure 2(f)). Since the optically created Te vacancy defects are permanent, the resulting phase transition is irreversible.

In another example, Kang et al. introduced Au nanoparticles onto MoS_2_ monolayer to induce a reversible plasmon-controlled 2H-1T phase transition [58]. Upon the decay of optically excited surface plasmons at the Au nanoparticles, hot electrons were injected into MoS_2_ layer (Figure 2(g)) to cause an unstable electron doping. The electron goes into the second orbital of Mo and creates an unstable excited state for Mo, which reduces the lattice stability of 2H phase MoS_2_ and drives a phase transformation. Raman spectra taken from MoS_2_ with and without Au nanoparticles after the light illumination were effective in revealing the 2H-1T phase transition (Figure 2(h)). The extent of phase transition could be effectively controlled by tuning the laser power for a given area of MoS_2_ flake, as the hot-electron injection rate from the Au nanoparticles depended on the incident light intensity. As the laser power decreases, a phase reversal occurred due to the loss of the hot electrons via diffusion made the doping density falls below the threshold for the 2H-1T phase transformation. This method possesses great potential in development of active devices requiring on-demand and transient phase transition.

## 4. Laser-Induced Oxidation of 2D Materials

With their tunable bandgaps, TMDCs are strong candidates for logical electronic devices. However, they do not have the enhanced electrical conductivity of graphene. Therefore, attempts were made to achieve bandgap engineering in graphene while retaining its high charge carrier mobility. One of the promising approaches is laser-induced oxidation of graphene. With an intermediate charge carrier mobility between pristine graphene and TDMCs, graphene oxide has a tunable bandgap. Reduction of graphene oxide would lead back to graphene.

**Figure 3:**
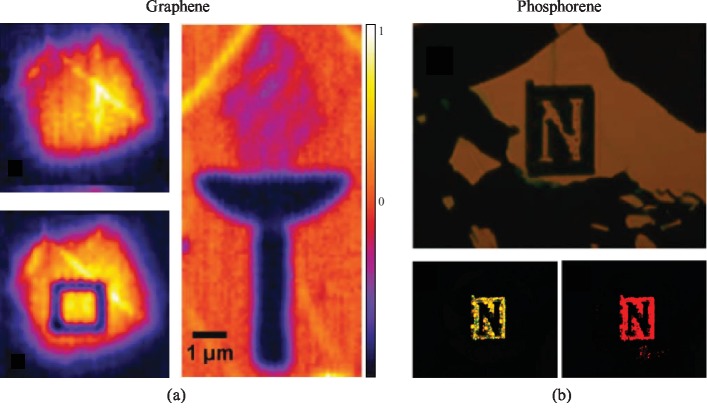
Laser-induced oxidation of 2D materials. (a) Left: four-wave mixing images of a suspended monolayer graphene before (top) and after (bottom) laser-induced oxidation. Right: four-wave mixing image of laser-patterned monolayer graphene on Si/SiO_2_. Reproduced from Ref. [60] with permission from the Royal Society of Chemistry, copyright 2015. (b) Optical image of letter “N” optically patterned on few-layer phosphorene (top) and four-wave mixing image of the same pattern at blue and green wavelength excitation (bottom). Reproduced from Ref. [64] with permission from the American Chemical Society, copyright 2015.

An initial attempt of opening bandgap in graphene exploits quantum confinement in the form of narrow graphene ribbons [59]. Recently, Aumanen et al. have demonstrated the tuning of electronic bandgap in graphene through laser-induced localized two-photon oxidation [60]. Oxidation was well controlled by altering the site and duration of laser-graphene interaction, leading to patterned graphene oxide regions within a graphene sheet. The electric properties were tuned in the patterned regions while graphene retained its high carrier mobility in the unpatterned regions. The characteristics of graphene oxide in the laser-irradiated regions were observed using both Raman mapping and four-wave mixing signals.  Figure 3(a) represents the four-wave mixing images of a suspended graphene sheet before (left-top) and three days after (left-bottom) patterning, as well as a patterned graphene on a Si/SiO_2_ substrate. The authors have further exploited the optical patterning of graphene via oxidation to realize a *p*-type FET where opening of bandgap arises due to laser-induced oxidation of the exposed graphene regions.

Like graphene, black phosphorus (BP) has also demonstrated great potential in applications requiring high carrier mobility [61]. However, implementation of BP has been challenging due to its poor optical response in the visible region [62] and low stability caused by ambient oxidation at room temperature [63]. Lu et al. have converted the BP oxidation into an advantage of optically tuning the bandgap of the 2D materials [64]. In their demonstrations, laser power was altered for controlled pruning and oxidation of multilayer BP at specific locations under ambient condition. These laser-induced flakes of phosphorene oxides and suboxides were fluorescent-active and demonstrated different colors when excited with different wavelengths. In contrast, pristine regions of the flake did not exhibit any photoactivity. The fluorescence of variable colors was due to the formation of a series of energy levels in the bandgap of phosphorene induced by the oxidation. The 2D material, when irradiated with lower-energy (longer-wavelength) light, excited the electrons to a lower energy level, which resulted in a fluorescence response having a relatively longer wavelength than the exciting light. This fluorescence phenomenon could be applied in the development of 2D multicolored displays.  Figure 3(b) (top) shows the optical image of a laser-pruned “N” pattern on the few-layer phosphorene. The bottom images of Figure 3(b) are from four-wave mixing signals of the same pattern, which shows different fluorescence colors when excited with blue and green wavelength lights, respectively. The multicolored display was extended to optically detect toxic ammonia gas based on the PL measurements, indicating the potential use of optically patterned BP in multifunctional photonic devices.

## 5. Direct Laser Writing of 2D Materials

Besides the laser-induced thickness-control procedures in Section 2, it is essential to develop methodologies that can thoroughly remove the selected parts of 2D materials to create patterns with specific shapes and sizes. Such patterned 2D materials are required for many solid-state devices such as light sources [65], light directors [66], and quantum emitters [67]. In this regard, both femtosecond and CW lasers have been successfully employed to directly pattern the 2D materials through laser ablation with a spatial resolution of under 200 nm.

### 5.1. Direct Femtosecond Laser Writing

Preliminary work on 2D material ablation was performed on graphene, where a high-powered femtosecond laser was utilized to convert the carbon to CO and CO_2_ and the optical patterning was imaged through upconversion luminescence [68]. With a donut-shaped laser beam that was generated through phase-shifting plates, one could abate multilayer graphene into nanodots and nanorods with a dimension of less than 200 nm. Later, Zhang et al. demonstrated optical patterning of monolayer graphene in both air and argon via femtosecond laser ablation [69]. The ablation in argon led to the narrower linewidths with the slightly higher laser fluence. Picosecond lasers were used to fabricate graphene ribbons via ablation at a speed of 0.25 m/s and a spatial resolution of the order of 10 *μ*m [70]. While these ablation methods worked on suspended graphene or graphene on glass substrates, an effective strategy must be developed to transfer the patterned 2D materials to any other substrates while retaining the structural integrity of the patterns for device applications. However, it becomes difficult to transfer monolayer patterns created by laser ablation due to heat-enhanced interactions of the monolayer with the substrate [39].

**Figure 4:**
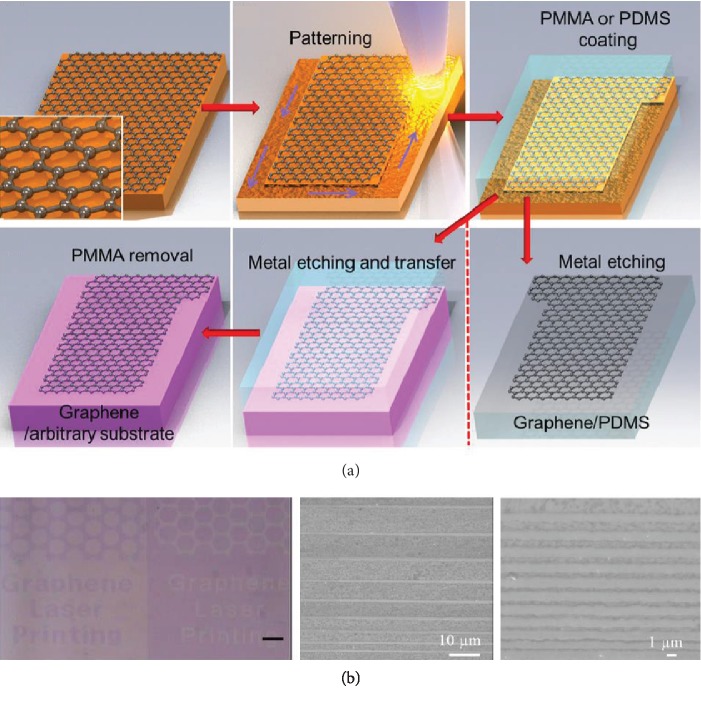
Femtosecond laser ablation of graphene. (a) Schematic of femtosecond laser ablation of graphene and subsequent transfer of the patterned graphene onto a PDMS or arbitrary substrate. (b) Optically patterned graphene transferred onto Si/SiO_2_ substrates. Scale bar in the left image: 100 *μ*m. Reproduced from Ref. [71] with permission from the American Institute of Physics, copyright 2012.

Park et al. demonstrated a procedure that involved laser ablation-based graphene patterning and subsequent transfer of the patterned graphene to an arbitrary substrate [71]. As illustrated in Figure 4(a), an as-grown graphene sheet on a metal substrate is patterned through ablation by a femtosecond laser beam. It is then coated with polydimethylsiloxane (PDMS) before the metal is etched away, resulting in the patterned graphene on the flexible PDMS substrate. To obtain patterned graphene on an arbitrary substrate, graphene upon laser ablation is coated with polymethyl methacrylate (PMMA) and transferred to the targeted substrate before removing PMMA. Figure 4(b) shows the optical image of transferred graphene patterns on a Si/SiO_2_ substrate (left panel), and the scanning electron micrographs of transferred graphene nanoribbons with variable widths ranging from 100 nm–9 *μ*m on Si/SiO_2_ substrates (middle and right panels). Despite its capability of achieving the multiscale and high-resolution graphene patterns on an arbitrary substrate, the ablation-and-then-transfer method has been limited using multiple wet chemical processes, which may cause graphene contamination and compromised pattern alignment.

To simplify the procedure, Yoo et al. have developed a single-step processing method that enables laser ablation-induced graphene patterning and simultaneous transferring to any substrate [72]. In this laser-induced pattern transfer, graphene is supported on a PMMA thin layer and then covered with a PMMA frame that features apertures for laser exposure. The stacked PMMA frame/graphene/PMMA thin layer is attached to a targeted substrate via vapor-assisted and surface tension-driven self-flattening. Through the apertures, a femtosecond laser beam is applied to ablate graphene along with PMMA thin layer into required patterns that are simultaneously transferred to the target substrate. The stacked PMMA frame/graphene/PMMA *thin layer* is peeled off, leaving the patterned graphene/PMMA thin layer on the substrate.

Although femtosecond laser ablation has been primarily demonstrated on graphene, it can potentially be employed to pattern many other 2D materials [73]. To achieve optimum performance in patterning the variable 2D materials, one needs to choose lasers with their power and wavelength matching the different bond strengths and optical responses.

### 5.2. Optothermoplasmonic Nanolithography

**Figure 5:**
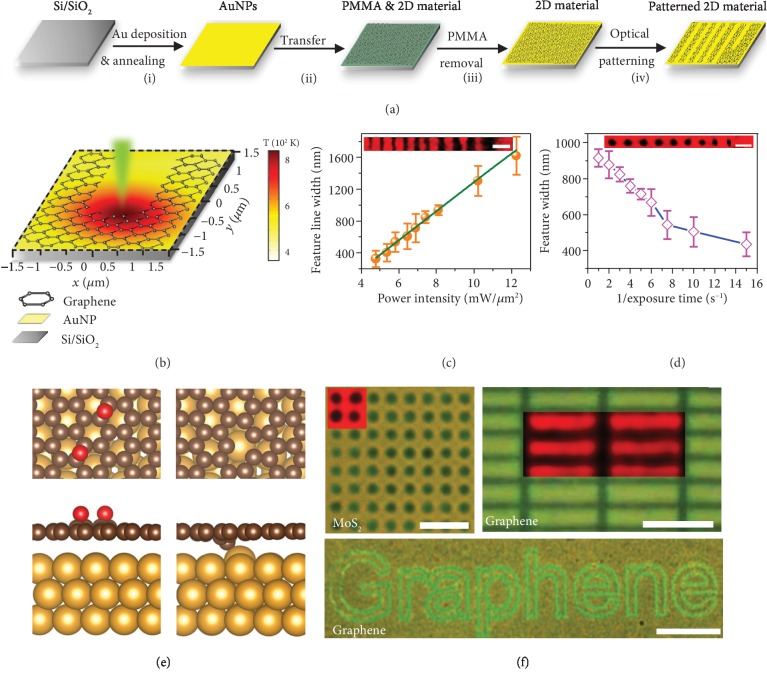
Optothermoplasmonic nanolithography. (a) Schematic for preparation and optical patterning of 2D material on a quasicontinuous Au film. (b) Schematic of optothermoplasmonic patterning of graphene along with simulated temperature distribution at the laser spot with an incident laser intensity of 6.4 mW/*μ*m^2^. (c) Linewidth of patterned graphene as a function of laser intensity. (d) Feature size of graphene nanoholes as a function of exposure time of laser. (e) DFT modelling of graphene oxidation on Au film. (f) Images of patterned MoS_2_ nanohole array (scale bar: 5 *μ*m), graphene nanorectangles (scale bar: 5 *μ*m), and “Graphene” text pattern (scale bar: 50 *μ*m). Reproduced from Ref. [74] with permission from Wiley, copyright 2018.

Lin et al. have developed optothermoplasmonic nanolithography that exploits plasmonic effects and CW laser-based ablation for low-power, high-throughput, and high-resolution patterning of 2D materials [74].  Figure 5(a) shows the schematic of the patterning process. Initially, Au is deposited and thermally annealed to form a quasicontinuous film, which serves as a thermoplasmonic substrate. 2D materials are then transferred onto the thermoplasmonic substrates. The sample is excited with a 532 nm laser for highly efficient photon-phonon conversion at the thermoplasmonic substrate, which results in localized thermal hotspots.  Figure 5(b) shows the simulated temperature increase of greater than 500 K for a low-power laser irradiation of 6.4 mW/*μ*m^2^, which enables localized ablation of 2D materials at the laser spot. The authors examined the feature size of patterned 2D materials under varying laser powers and exposure times (Figures 5(c) and 5(d)). Density functional theory (DFT) calculations were performed to understand the catalytic effects of Au film on graphene patterning (Figure 5(e)). This technique is versatile for creating arbitrary patterns in a variety of 2D materials such as graphene and TMDCs (Figure 5(f)). The patterned 2D materials can be readily transferred to any other substrates.

## 6. Applications of Optically Patterned 2D Materials

The diverse optical patterning techniques presented in this review show potential prospects in several nanoscale applications. In this section, we select and analyze a few applications that are based on the optically patterned 2D materials.

**Figure 6:**
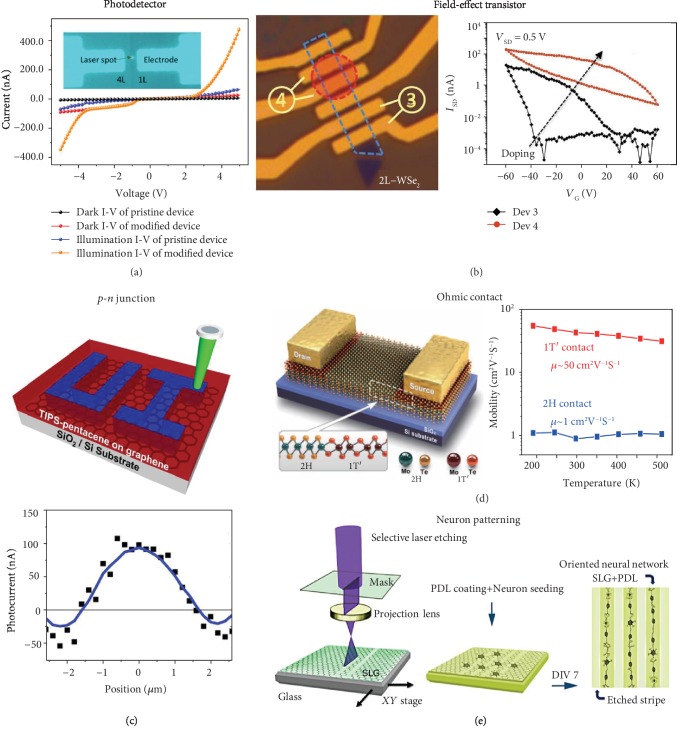
Applications of optically patterned 2D materials. (a) I-V characteristics of photodetectors based on pristine and laser-thinned multilayer MoS_2_ under dark and illumination conditions. Reproduced from Ref. [75] with permission from the American Chemical Society, copyright 2014. (b) Field-effect transistors based on undoped and phosphorus-doped WSe_2_. In the left image, the blue dashed box outlines the bilayer WSe_2_ flake and the red dashed circle denotes the laser-irradiated area that exhibits the doping effect. Reproduced from Ref. [52] with permission from Wiley, copyright 2015. (c) Schematic and photoresponse of graphene-based *p*-*n* junction fabricated by laser-controlled oxidation. Reproduced from Ref. [81] with permission from the American Chemical Society, copyright 2014. (d) Left: schematic of a 2D transistor with laser-induced heterophase homojunction of 2H and 1T′ phases in MoTe_2_. Right: measured carrier field-effect mobility in the devices with 1T′-MoTe_2_/metal contact and conventional 2H-MoTe_2_/metal contact, respectively, as a function of temperature. Reproduced from Ref. [56] with permission from the American Association for the Advancement of Science, copyright 2015. (e) Schematic of oriented neural network evolution on optically patterned single-layer graphene (SLG) on a glass substrate coated with poly-D-lysine (PDL). DIV 7: 7 days in vitro. Reproduced from Ref. [82] with permission from the Nature Publishing Group, copyright 2013.

Laser thinning has the potential to create functional devices with spatially varying step morphology of 2D materials involving different layer thicknesses at different positions. Utilizing this capability, functional 2D junctions with spatially varying bandgaps (due to different thicknesses) were fabricated, which enabled a photodetector with enhanced performance [75].  Figure 6(a) shows the I-V characteristics of photodetectors based on pristine and laser-thinned multilayer MoS_2_ under on and off illumination. The inset shows two distinct thicknesses of the 2D materials in the device, which enabled a built-in potential barrier for a better photodetection performance. Also, 2D material properties significantly change with the layer thickness. Graphene exhibits a decreasing thermal conductivity as the number of layers increases [17, 76]. By implementing the site specificity of optical thinning methods, *in situ* thickness control can be achieved, thereby achieving perfect thermal conductance for varying cooling needs within the microdevice as a heat sink. Similarly, TMDCs transform to direct-bandgap semiconductors and present strong photoluminescence while going down towards monolayer thickness [45]. In addition, it has been reported that dielectric constant of 2D material could be changed by varying the thickness of the 2D material [77] for applications in FETs [78], topological insulators [79], and pulsed laser generation [80].

Many 2D applications in electronic and optoelectronic devices such as photodetectors and FETs require the capability of tuning charge carrier type and density of the 2D materials to achieve *p*-type or *n*-type doping with high site selectivity and high stability. This can be achieved by laser-induced doping of the 2D materials. As shown in Figure 6(b), laser-exposed FET junction based on WSe_2_ bilayer exhibits an increased source-drain current as a function of back-gate voltage by four orders [52]. The increased conductance as well as shift of threshold voltage indicates that the laser-induced phosphorus doping enhances the *p*-type characteristics of the WSe_2_. As another example, Figure 6(c) shows the schematic and photoresponse of *p*-*n* junctions in graphene, which are fabricated via direct-laser-writing-induced spatially modulated doping in graphene covered by a pentacene thin film [81]. Photooxidation of pentacene transformed initially *p*-type graphene to *n*-type at the laser-irradiated sites, which enabled the creation of *p*-*n* junctions on graphene with excellent photoresponses.

A good electrical contact to the semiconducting 2D material is critical to high-performance 2D electronics. Yang and coworkers exploited laser-induced phase transition of MoTe_2_ to solve the issue of Schottky contact between the semiconducting 2D channel materials and the metallic electrodes. Briefly, a laser beam is imposed on the surface of mechanically exfoliated flake of multilayer MoTe_2_ in 2H phase to induce phase transition from semiconducting 2H phase to metallic 1T′ phase in the desired area where metal electrode will be located (Figure 2(d) and Figure 6(d)) [56]. Figure 6(d) (left) illustrates a transistor where such a laser-induced 2H-1T′ phase transition creates heterophase homojunction structure in MoTe_2_ and thus ohmic contact between the metallic electrode and the 2D material. As shown in Figure 6(d) (right), the carrier field-effect mobility in the device with 1T′-MoTe_2_/metal contact is enhanced by a factor of ~50 compared with that in the device with a conventional 2H-MoTe_2_/metal contact.

Besides their electronic and optoelectronic applications relevant to information technology, 2D materials are finding their uses in biology and life sciences. For example, 2D materials with high electrical conductivity act as charge-sensitive surfaces that are ideal for biomolecular sensing. Lorenzoni and coworkers employed optically patterned graphene on substrates to study biological neural networks during their evolution. They observed high alignment of neuron adhesion and growth on the patterned substrate as illustrated in Figure 6(e) [82]. These low-cost functionalized graphene patterns are stable against rinsing and can be used repetitively for studying the evolution of neurons.

Apart from electronic and biological applications of patterned 2D materials, many photonic applications have been studied in the recent years [83, 84]. Graphene nanopatterns, for instance, exhibit tunable plasmonic resonance from midinfrared to radio waves, which could be used in several photonic applications such as modulators, polarizers, and enhanced IR spectroscopy [22]. Ju et al. experimentally demonstrated a tunable response for graphene in the terahertz frequency (far infrared) range by optically patterning graphene ribbons with width varying between 1 *μ*m and 4 *μ*m [85]. 2D materials were also developed for single-photon quantum emitters [86] and lasers [65, 87]. The future of 2D materials holds the integration of such photonic applications to be inscribed onto lab-on-chip technologies where high-resolution patterning is required [88]. Meanwhile, optical patterning methodologies with the resolution as low as 200 nm make them promising approaches for pattering 2D materials and further enhancing the functionality of photonic applications.

In summary, these selected application examples illustrate that laser-mediated patterning methods can not only improve the performances of the conventional 2D devices such as photodetectors and FETs but also induce fascinating phenomena for development of new applications in cellular biology, photonics, and neuroscience. Many reports have been published solely on the applications of 2D materials [89–91] to which site-specific on-demand optical patterning can be readily applied to enhance the performances or introduce new functionalities.

## 7. Perspective

Despite the advances discussed herein, we realize that there is an enormous scope for further development directed towards universal fabrication and patterning techniques for 2D materials. Several opportunities and challenges remain in improving optical patterning in terms of precision, resolution, surface roughness, versatility, diversification, and execution. One of the most prominent limitations of optical patterning is attaining precision or resolution below the diffraction limit of light. To improve the resolution of optical patterning, one-dimensional-phase (1D-phase) shifting plates have been utilized to overcome this limitation; the resolution of optically patterned 2D materials is still of the same order [68, 92]. Spatial light modulators can be utilized by incorporating laser beam phase change at each pixel to focus the beam much below the diffraction limit [93, 94]. Engineering of plasmonic substrates to focus optothermal nanohotspots into prescribed geometry could also be the key for patterning 2D materials at sub-100 nm resolution. Apart from visible and IR radiation, ultraviolet (UV) rays have also been used for more than 15 years to pattern microelectronics with higher resolutions due to the much shorter wavelengths. Deep UV rays with a wavelength of 193 to 248 nm have achieved a resolution down to sub-100 nm [95]. For instance, Yin et al. have implemented UV radiation to fabricate nanochannels with width and height less than the diffraction limit of the laser beam [96], which can be extended to controlled patterning of 2D materials. Later, extreme UV radiation (wavelength~13 nm) has been employed to fabricate microelectronics at a limit of sub-50 nm resolution [97] through the interference of multiple beams. However, utilization of photomasks and photocurable polymers along with high absorption of UV radiation by optical elements in the pathway limits the implementation in 2D materials. With application of UV rays, coupled with further optimization of laser power and optical pathway, a higher resolution could be achieved, which would promote optical patterning of 2D materials towards on-demand and *in situ* on-chip fabrication of functional nanodevices.

Other methods such as tip-based scanning probe lithography techniques [98, 99] and block copolymer lithography [100] have been put into effect to pattern 2D materials to reduce the resolution limits. Although scanning probe lithography has achieved resolution as low as 10 nm [101], the scalability is a disadvantage due to the serial operation and direct contact nature of the patterning method. Block copolymer lithography has been realized to pattern samples as large as 2 mm × 2 mm while achieving 36 nm resolution [102]. However, this is a multistep process, which works for periodic patterns and results in the contamination of the samples. Moreover, achieving the highest resolution of these techniques requires tedious multiparameter optimization [103]. Single-step optical patterning techniques provide the flexibility and higher throughput that are unavailable in these conventional patterning methods. Moreover, the use of spatial light modulators or digital micromirror devices could split a single laser beam into multiple independent beams [104, 105] to further increase the scalability.

So far, optical patterning technologies have mainly been focused on homogeneous 2D materials. Recent reports show the immense potential of van der Waals heterostructures of 2D materials in energy storage [106], memory devices [107], and plasmonics [108]. However, to implement these 2D heterostructures in practical applications, precise three-dimensional control over their geometry and composition is required, which can hardly be achieved by the existing optical patterning techniques. Advanced optical patterning of the heterostructures will require temporal and spatial exploitation of the variable material-specific mechanisms in a single system where laser beams of different wavelengths, powers, and durations can be applied depending on composition and position of any targeted 2D layer. One technical approach is to implement a feedback mechanism to detect the photoresponse of individual 2D layers and to automate parametric control on laser power, wavelength, and position with an intelligent system. Several *in situ* measurement techniques based on optical imaging and spectroscopy can be used as a feedback mechanism to control the working laser parameters, as already used in colloidal manipulation and assembly works [109–112]. Spatial light modulators and digital micromirror devices have been used to control the laser beam position [104, 113] and to reshape the laser beam to achieve single-shot, high-throughput, and high-resolution patterning. Furthermore, a standardized methodology to determine a universal figure-of-merit of patterning accuracy through postprocessing of images could be established [114].

## 8. Summary

Optical patterning techniques have played a vital role in the tremendous growth of scientific research and applications based on 2D materials. Such techniques enable various physical and chemical changes induced precisely at the required sites of the 2D materials to create functional nanostructures and devices. With their advantages of tunable laser intensity, tunable laser wavelength, and site specificity, optical patterning holds the key to attain versatile high-throughput fabrication of 2D materials with submicron features. Further studies in this field would enable optimized, on-demand and universal fabrication of heterogenous 2D materials with feature sizes down to atomic scale, marking a significant advance in nanoscience and nanoscale industrial revolution.
